# Management of Postsurgical Complication in Multiple Implant-Infected Postextraction Sites in the Lower Arch

**DOI:** 10.1155/2020/8869046

**Published:** 2020-09-29

**Authors:** Frank Mayta-Tovalino, José Rosas, Cesar Mauricio-Vilchez, Silvia Luza, Daniel Alvitez-Temoche, Franco Mauricio

**Affiliations:** ^1^Faculty of Health of Sciences, Postgraduate Department, Universidad Cientifica Del Sur, Lima, Peru; ^2^Faculty of Dentistry, School of Dentistry, Universidad Nacional Mayor de San Marcos, Lima, Peru; ^3^Postgraduate Department, Faculty of Stomatology, Universidad Peruana Cayetano Heredia, Lima, Peru; ^4^School of Dentistry, Academic Department, Universidad Privada San Juan Bautista, Lima, Peru; ^5^Faculty of Dentistry, Academic Department, Universidad Nacional Federico Villarreal, Lima, Peru

## Abstract

Currently, dental implants are a very frequent therapeutic alternative for replacing missing teeth. However, they are not exempt from developing complications of infectious origin. Therefore, this case report describes a 67-year-old female patient presenting infectious complications caused by suture rupture. Surgery combined with therapeutic management with antibiotics was performed, allowing preservation of the osseointegration of the implants in the lower arch. Within the limitations of this study, it was shown that more research is needed to determine the success and survival of implants presenting complications due to infections during the osseointegration process.

## 1. Introduction

Currently, the indication of dental extraction is very common before the placement of dental implants, especially when these teeth have a reserved prognosis. However, one of the main problems of dental extraction is that it usually significantly affects the volume of soft and hard tissues after tooth extraction. This tissue loss can directly affect the future positioning of the implants causing poor prosthetic rehabilitation [1, 2].

Postextraction implant placement mainly has a social and clinical impact since the patient usually recovers esthetic, phonetic, and chewing components faster, significantly reducing the treatment time and avoiding a second surgical intervention [1]. Several studies have shown that immediate placement of implants in fresh extraction areas is successful when appropriate protocols are carried out. However, implant placement in infected areas is considered a risk factor, although data from animal and human studies have shown similar success rates for implants placed in infected sites compared to uninfected sites [2, 3]. Otherwise, immediate posttooth implants are usually indicated to replace teeth lost due to injuries or infections of chronic origin, for example, teeth with a history of failed endodontic treatment [3–5].

On the other hand, the success of dental implant placement in sites with and without localized infection is different regardless of the pre- and postoperative administration of systemic antibiotics. Indeed, it has been reported that dental implant placement in infected sites increases the potential risk of unsuccessful osseointegration of the implant [4–8].

Thus, the aim of this case report was to present the management of postsurgical complications in multiple implant-infected postextraction sites in the edentulous lower arch.

## 2. Case Presentation

**Figure 1:**
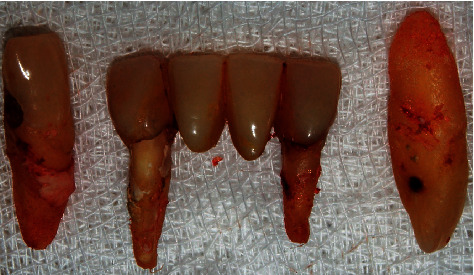
Atraumatic dental extraction.

A 67-year-old female patient with a history of high blood pressure and controlled diabetes consulted with the aim of undergoing the placement of hybrid prostheses on dental implants in the jaw similar to a previously placed prosthesis in the upper jaw. The patient was completely edentulous in the upper and lower arch, having only teeth 32, 33, 42, and 43 and a bridge in poor condition with pillars in teeth 32 and 42. She also presented Miller's Grade 3 mobility and root carious lesions in teeth 33 and 43 (Figure 1).

**Figure 2:**
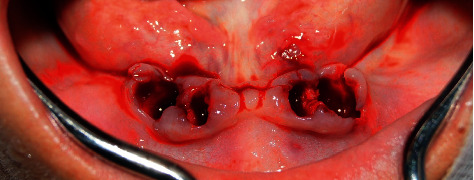
Fresh postextraction sockets.

Postextraction implant placement was planned in the anteroinferior area of the mandible for subsequent oral rehabilitation with a hybrid prosthesis. Based on a previous tomographic diagnosis, atraumatic tooth extraction was performed with the help of periotome curettes PT2 and PT3 (Hu-Friedy) to maximize alveolar bone integrity. Syndesmotomy was then performed with the help of straight drills (Hu-Friedy) (Figure 2).

**Figure 3:**
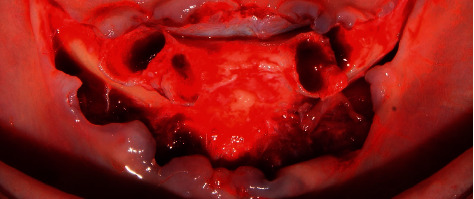
Fresh postextraction sockets.

**Figure 4:**
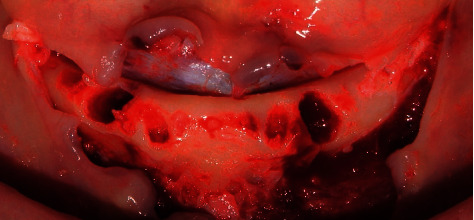
Crestal bone remodeling.

**Figure 5:**
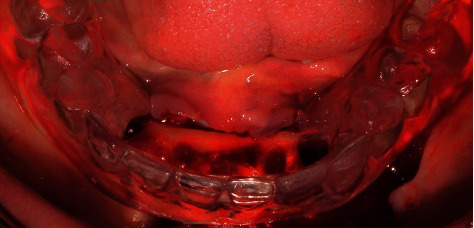
Adaptation of the surgical guide prior to the placement of dental implants.

**Figure 6:**
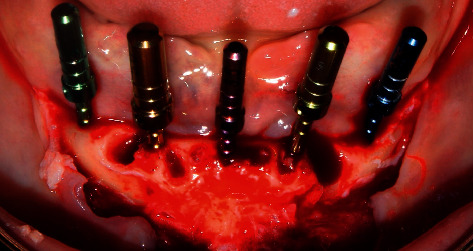
Test of parallelism of dental implants.

**Figure 7:**
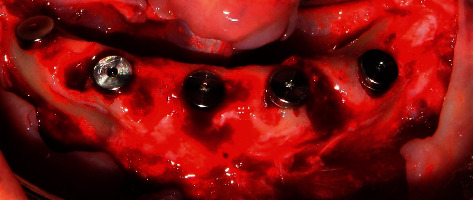
Postextraction implant placement.

Subsequently, a crestal flap was raised at the interforaminal level from quadrants III to IV under 2% lidocaine anesthesia with the aid of Prichard PPR3X and P24G curettage (Hu-Friedy). Due to the presence of multiple bone spicules, the crestal bone was then reshaped with the help of HM251SX tungsten carbide surgical drills and a low-speed micromotor with abundant saline solution to avoid necrotizing bone tissue (Figure 3). Thereafter, a surgical guide was placed on the lower jaw area to locate the site for surgical drilling for the dental implants. The surgical drilling protocol involved 4.3 × 13 mm NeodentTitamax II Plus implants (Curitiba, Brazil) for all implants. Drilling was started with the lance, Titamax 2.0 drill, 2/3 pilot drill, Titamax 3.15 drill, 3/3.75 pilot drill, and finally with a countersink II 3.8 drill (Figures 4–7).

**Figure 8:**
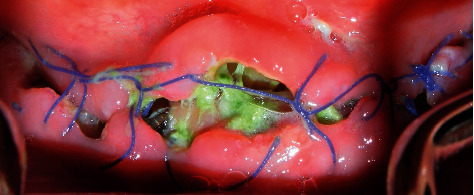
Infectious process with rupture of the suture material.

**Figure 9:**
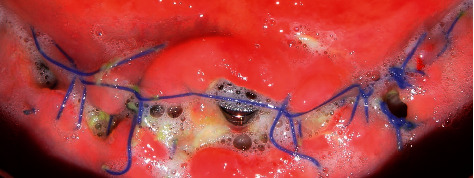
Surgical cleaning of the implant area.

**Figure 10:**
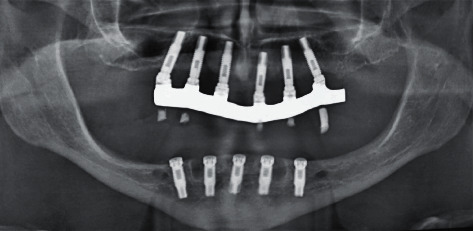
Control radiograph at 30 days.

**Figure 11:**
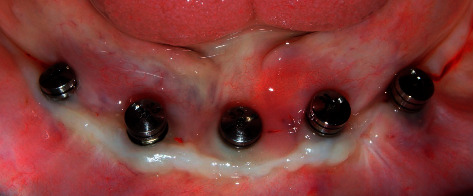
Tissue stabilization at 6 months.

Following the initial steps, the patient was prescribed prophylactic antibiotic treatment (1 g of amoxicillin, 1 hour before surgery followed by 500 mg twice a day for 7 days). However, on the fifth day of follow-up, clinical examination showed the rupture of the polyglycolic acid suture, triggering an infectious process at the level of all the implants. Traces of food were evident with a purulent exudate at the crestal bone level (Figure 8). According to the protocol, surgical cleaning was first immediately performed with chlorhexidine 0.12% and cethylpyridinium chloride 0.05 (Perio • Aid®). Second, the suture was removed, and new suturing was carried out. Clinical management included clindamycin 600 mg/4 ml (Dalacin®) every 24 hours for 4 days plus dexamethasone 4 mg/2 mg (Fortecortin®) every 12 hours for 3 days (Figure 9). The follow-up consisted of periodic control consultations per week for one month. Follow-up panoramic radiography (Figure 10) showed fresh alveoli healing favorably. Finally, after 6 months of follow-up, the healing abutments were placed with correct adaptation of soft tissues allowing preparation of the hybrid prosthesis (Figure 11).

## 3. Discussion

Several studies have recently described the impact of antibiotic administration on the survival rate of dental implants. It has been suggested that the application of local instead of systematic antibiotics might cause fewer complications, such as allergic reactions and resistance to antibiotics, among others. However, the use of chlorhexidine is also important to avoid possible postextraction irrigation, with studies supporting the effectiveness of chlorhexidine against multiple bacterial strains inducing periodontal and endodontic infections of the oral cavity. Some studies have suggested that the preoperative use of mouthwash with chlorhexidine significantly reduces the risk of surgical complications of the implant due to bacterial infection by significantly reducing viral and bacterial load, especially in hard and soft tissues such as bone, periodontal ligament, and root canals, among others [4, 9].

However, it should be mentioned that most research on the immediate placement of implants after extraction is linked to the preservation of esthetic conditions, although this is less important in the posterior region [10]. Alveolar infection in the posterior sector is known to be a risk factor for dental implant failure. Several recent studies have shown that the postextraction implant technique in infected areas has a significantly increased risk of osseointegration failure compared to implants conventionally placed in healthy sites such as in this case report [11, 12].

Despite the development of an infectious process during follow-up, the dental implants achieved optimal primary stability >40 N/cm, and no further complications placed osseointegration at risk. Similar to the results of other studies, this was evident even at 6 months of follow-up [13, 14]. Although postextraction implant placement has been widely validated, few studies have reported results at infected sites during the healing process. Some studies have described the use of erbium, chromium-doped yttrium, scandium, gallium, and garnet (Er,Cr : YSGG) laser therapy to disinfect infectious processes, which can reduce treatment time [15].

It is important to recognize that periodontitis is a highly prevalent inflammatory disease produced by periodontal bacteria, which causes the destruction of support tissues that induces tooth loss. Therefore, this may be a risk factor for the failure of future dental implants [16]. In addition, the nutraceutical agent has been shown to be effective in reducing inflammatory levels in patients with periodontal disease. It is important to recognize the importance of certain mediators such as ADMA (asymmetric dimethylarginine), malondialdehyde, and vitamins as risk factors for the presence of periodontitis and as a cause of tooth loss. [17, 18].

Finally, the results of this case report show that the placement of implants in infected sites immediately after tooth extraction for periodontal or endodontic reasons is a safe option [7]. The implant survival rates ranged from 92% to 100% in sites with endodontic infection. Taking into account the limited and heterogeneous scientific evidence available in this regard, larger and more rigorous studies are required to evaluate the effectiveness of this treatment [11].

## 4. Conclusion

Prompt and timely management of surgical complications in dental implant placement can preserve osseointegration. It is recommended to accurately identify the site and the etiology of infection in order to perform adequate treatment.
